# Phytosynthesis of Palladium Nanoclusters: An Efficient Nanozyme for Ultrasensitive and Selective Detection of Reactive Oxygen Species

**DOI:** 10.3390/molecules25153349

**Published:** 2020-07-23

**Authors:** Ravi Mani Tripathi, Sang J. Chung

**Affiliations:** 1 School of Pharmacy, Sungkyunkwan University, 2066 Seoburo, Jangan-gu, Suwon, Gyeonggido 16419, Korea; rmtripathi02@gmail.com; 2 Amity Institute of Nanotechnology, Amity University Uttar Pradesh, Sector 125, Noida 201303, India

**Keywords:** palladium nanoclusters, nanozyme, peroxidase mimetic activity, colorimetric detection, hydrogen peroxide, 3,3′,5,5′-tetramethylbenzidine

## Abstract

Hydrogen peroxide is a low-reactivity reactive oxygen species (ROS); however, it can easily penetrate cell membranes and produce highly reactive hydroxyl radical species through Fenton’s reaction. Its presence in abnormal amounts can lead to serious diseases in humans. Although the development of a simple, ultrasensitive, and selective method for H_2_O_2_ detection is crucial, this remains a strategic challenge. The peroxidase mimetic activity of palladium nanoclusters (PdNCs) has not previously been evaluated. In this study, we developed an ultrasensitive and selective colorimetric detection method for H_2_O_2_ using PdNCs. An unprecedented eco-friendly, cost-effective, and facile biological method was developed for the synthesis of PdNCs. This is the first report of the biosynthesis of PdNCs. The synthesized nanoclusters had a significantly narrow size distribution profile and high stability. The nanoclusters were demonstrated to possess a peroxidase mimetic activity that could oxidize peroxidase substrate 3,3′,5,5′-tetramethylbenzidine (TMB). Various interfering substances in serum (100 μM phenylalanine, cysteine, tryptophan, arginine, glucose, urea, Na^+^, Fe^2+^, PO_4_^3−^, Mn^+2^, Ca^2+^, Mg^2+^, Zn^2+^, NH_4_^+^, and K^+^) were included to evaluate the selectivity of the assay, and oxidation of TMB occurred only in the presence of H_2_O_2_. Therefore, PdNCs show an efficient nanozyme for the peroxidase mimetic activity. The assay produced a sufficient signal at the ultralow concentration of 0.0625 µM H_2_O_2_. This colorimetric assay provides a real-time, rapid, and easy-to-use platform for the detection of H_2_O_2_ for clinical purposes.

## 1. Introduction

Enzymes are frequently used to catalyze the conversion of biomolecules; however, they require mild conditions to facilitate the reaction. In contrast, chemical catalysts can easily facilitate reactions under harsh conditions, such as high pressures, high temperatures, organic solvents, and extreme pH [1,2]. Enzymes are extensively used in industry, medical, and biological fields owing to their substrate specificity and high catalytic activities. However, enzymes have some inherent limitations, such as high preparation and purification costs, low operational stability, sensitivity to environmental conditions, and difficulties in recycling and reuse [3]. To overcome these limitations, researchers are exploring the possibility of developing artificial enzyme mimics that are cost-effective and have high stability. Several studies have explored the development of artificial enzyme (also called nanozyme [3]) mimic materials, such as ZnO-Pd nanosheets [4], Fe3O4 nanoparticles [5], Cu–Ag on graphene oxide [6], and metal–organic frameworks [7]. These nanozymes have enzyme mimetic activity and are widely used for the oxidation of peroxidase substrate 3,3′,5,5′-tetramethylbenzidine (TMB) for the detection of H_2_O_2_.

The three main reactive oxygen species (ROS) are hydrogen peroxide, superoxide, and hydroxyl radicals, which are normal products of cellular metabolism [8]. H_2_O_2_ is a low-reactivity molecule; however, it can actively penetrate cell membranes and produce the most reactive form of oxygen, that is, hydroxyl radicals, through Fenton’s reaction (H_2_O_2_ + Fe^2+^ → Fe^3+^ + OH^−^ + OH•) [9]. An abnormal production of H_2_O_2_ can lead to oxidative stress, which is the main contributor to aging [10] and leads to serious diseases such as diabetes [11], cancer [10], and neurodegenerative Alzheimer’s and Parkinson’s diseases [12,13]. Furthermore, H_2_O_2_ is a potential molecule for various clinically important applications. The most widespread use of H_2_O_2_ is for the oxidation of peroxidase chromogenic substrates to detect biological molecules. Hence, it is important to develop not only an effective nanozyme to replace the enzyme, but also a highly sensitive and selective method for the detection of H_2_O_2_. This provides the motivation for developing an effective nanozyme for peroxidase mimetic activity as well as a highly sensitive and selective colorimetric detection method for H_2_O_2_. We previously developed an ultrasensitive and selective colorimetric detection method for lead [14]. We previously reported the biosynthesis of triangular and hexagonal gold nanoparticles [15], palladium nanoparticles [16,17,18], and HAp nanofibers [19]. The present study reports the biosynthesis of nanoclusters.

In this study, we developed an eco-friendly, simple, and cost-effective method for the synthesis of palladium nanoclusters (PdNCs) using phytochemicals (plant extracts). To the best of our knowledge, no nanoclusters have been exploited for peroxidase mimetic activity thus far. Furthermore, we have not found previous reports on the use of PdNCs as nanozymes. The biosynthesized nanoclusters exhibited ultrasensitive and selective detection of H_2_O_2_. The novelty of the reported method for the detection of H_2_O_2_, in comparison with previously reported methods, is presented in a tabulated form in Table 1 [20,21,22,23,24,25,26,27,28,29,30,31,32,33]. The biosynthesis of palladium nanoclusters in itself is a novel achievement, and no reports are available on the use of biological methods for this purpose.

## 2. Results and Discussion

### 2.1. UV/Vis Analysis

**Figure 1:**
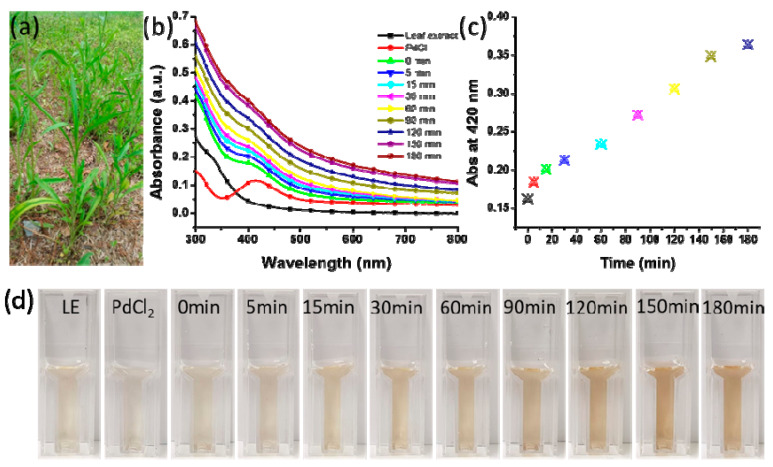
Biosynthesis of palladium nanoclusters (PdNCs): (**a**) *Erigeron canadensis* L.; (**b**) UV/Vis spectra of the leaf extract, palladium chloride, and biosynthesis of PdNCs; (**c**) Absorbance at 400 nm as a function of time; (**d**) The impact of incubation time on the color of reaction mixture (LE: leaf extract).

The biosynthesis of PdNCs was determined by measuring their absorbance as a function of time after the addition of leaf extract to the palladium chloride solution. *Erigeron Canadensis* L. leaf extract was used as the reducing and capping agents for the synthesis of nanoclusters (Figure 1a). The peak observed at 420 nm for the palladium chloride aqueous solution indicated the presence of Pd^2+^ ions. This peak almost disappeared after the synthesis of the nanocluster (Figure 1b) and the amount of PdNC was estimated based on the amount of the used PdCl_2_. The formation of nanoclusters was monitored at different time intervals (0, 5, 15, 30, 60, 90, and 180 min) by scanning the UV/Vis absorbance spectra of the samples. Figure 1d shows the impact of the incubation time on the color of the reaction mixture. As soon as the leaf extract was added to the aqueous solution of palladium chloride, the color intensity increased until an incubation time of 150 min. However, after 180 min of incubation, no color change was observed visually. The UV/Vis spectra show that the absorbance increased with increasing incubation time, but the increase was minimal after 180 min compared with that at 150 min (Figure 1b). Changes in the absorbance spectra at 400 nm as a function of time and stationary phase were observed after 150 min (Figure 1c). Therefore, the optimum reaction time was found to be 150 min.

### 2.2. Dynamic Light Scattering (DLS) Analysis

The synthesized nanoclusters were analyzed by DLS to determine the size distribution profile. The size distribution results indicate that the nanoclusters had a Z-average diameter of 64.47 nm (Figure S1). Particles sized <100 nm are considered useful for various applications owing to their surface-to-volume ratios. In addition, particles of <100 nm can effortlessly cross the plasma membrane cells for different types of applications. The stability of nanoparticles is also an important consideration for various applications. The polydispersity index is dimensionless and scaled such that values smaller than 0.05 are generally observed with highly monodisperse standards, whereas those greater than 0.7 show that the sample has a significantly wide particle size distribution [34]. The synthesized nanoclusters exhibited a polydispersity index of 0.053, indicating monodispersion. It has been reported that the polydispersity index must be less than 0.7 for good quality nanomaterials [35].

### 2.3. Transmission Electron Microscopy (TEM) Analysis

**Figure 2:**
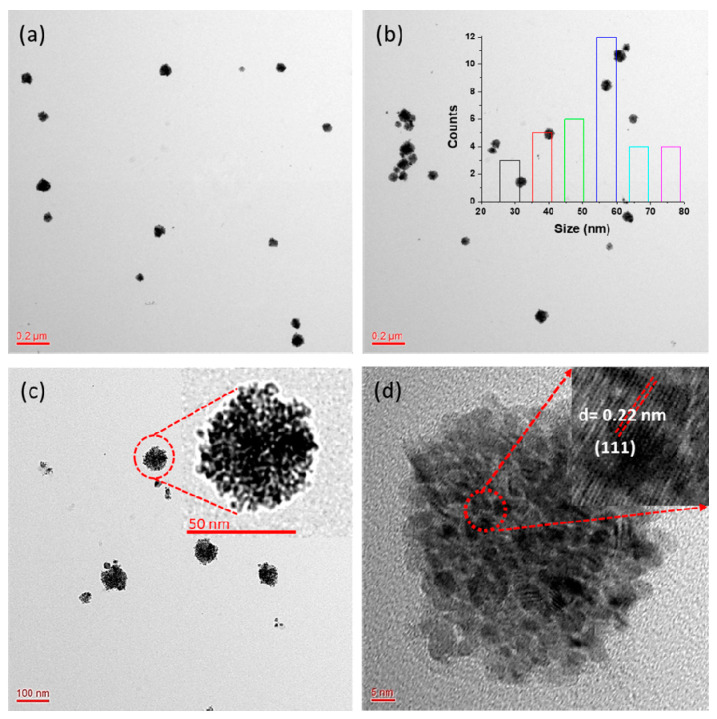
Transmission electron microscopy (TEM) micrographs of the PdNCs at different magnifications: (**a**) Wide scan of the sample at 0.2 µm for an overall view; (**b**) Uniformity of the nanoclusters at low magnification, the inset shows the nanoclusters size distribution histogram; (**c**) Scan focused on a small area of the sample in which the inset shows a zoomed-in view of a single nanocluster; (**d**) High resolution (HR)-TEM image showing the crystalline nature of the nanoclusters in which the inset shows the inter-atomic spacing (d-spacing).

The morphology, size, and crystalline nature of the synthesized PdNCs were determined using TEM. Various magnifications were used to obtain the micrographs to better elucidate the nature of the nanoclusters (Figure 2). The Z-average diameter of the synthesized nanoclusters was determined to be 64.47 nm in the DLS analysis (Figure S1), whereas the TEM analysis indicated a diameter of approximately 57 nm, because the DLS showed the hydrodynamic size of the nanostructures. The TEM micrographs show that the synthesized nanoclusters had a significantly narrow size distribution of 28–76 nm (Figure 2a,b). The size distribution histogram shows that the synthesized clusters have a significantly narrow size distribution, ranging from 28 to 76 nm. The histogram also shows that the average nanocluster belongs to 57 nm (inset in Figure 2b). The inset in Figure 2c shows an enlarged image of a single nanocluster, revealing that significantly small particles combined to form the nanoclusters. The high-resolution TEM (HR-TEM) results show that the nanoclusters exhibited the lattice-fringe characteristic of crystalline materials. The inset of Figure 2d shows these lattice fringes, clearly revealing the crystalline nature of the nanoclusters; the inter-atomic spacing (d-spacing) was determined to be 0.22 nm. Few reports have been published for the chemical synthesis of PdNCs, but all of these syntheses required chemicals that increase the production cost as well as create environmental problems [36,37]. The method proposed in this study is simple, rapid, cost-effective, environmentally friendly, and requires no hazardous chemicals. Furthermore, the results show that the developed method could successfully synthesize nanoclusters of good quality. The nanoclusters were characterized three months after synthesis, and no agglomeration/aggregation was observed, confirming the stability of the nanoclusters. The stability of the nanoclusters was further analyzed by sonicating the colloidal solution of nanoclusters, and the resulting TEM micrographs showed no notable influence of mechanical shaking on the nanoclusters (Figure S2). DLS analysis was done after sonication of as-synthesized nanoclusters. The Z-average of as-synthesized nanoclusters is found to be 64.47 nm. After sonication, the Z-average changes to 67.44 nm with broad peaks (Figure S3). Therefore, sonication has significantly little impact on the size of nanoclusters, which shows that the synthesized nanoclusters are stable.

### 2.4. Selected Area Electron Diffraction (SAED) and X-Ray Diffraction (XRD) Analysis

**Figure 3:**
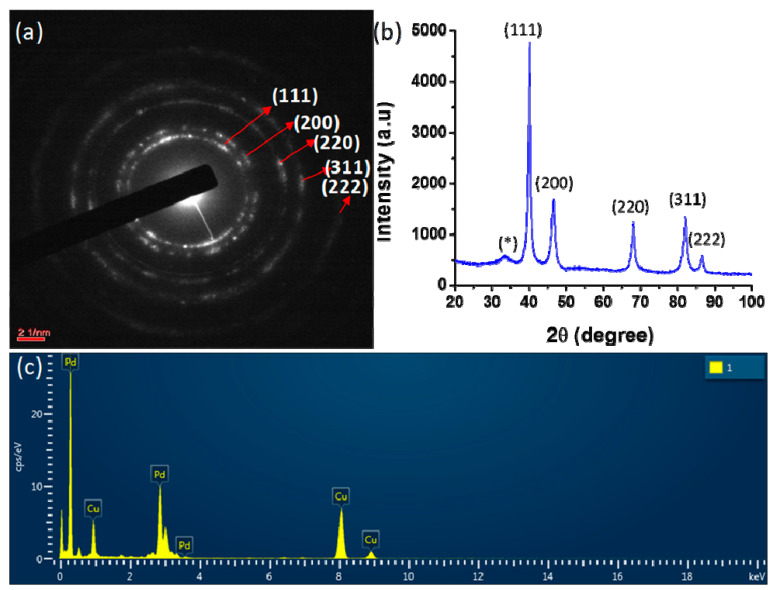
Biosynthesized PdNCs: (**a**) Selected area electron diffraction (SAED) pattern showing the presence of rings corresponding to the crystalline nature of nanoclusters; (**b**) X-ray diffraction (XRD) pattern (* the reflections due to residual moieties of the leaf extract); and (**c**) Energy-dispersive X-ray spectroscopy (EDX) spectrum exhibiting the signal for elemental palladium.

The selected area electron diffraction (SAED) pattern of the synthesized PdNCs shows the crystalline nature of nanoclusters. The SAED results also indicate the presence of rings corresponding to the (111), (200), (220), (311), and (222) planes of palladium with a face-centered cubic structure (Figure 3a). These planes were further analyzed by XRD using powdered samples of PdNCs obtained by freeze drying the colloidal solution. The XRD pattern of the PdNCs exhibited distinct reflections at 2θ of 40.05°, 46.59°, 68.10°, 82.03°, and 86.59°, which corresponded to the (111), (200), (220), (311), and (222) lattice planes of a face-centered cubic (fcc) lattice, respectively (Figure 3b). These reflections are characteristic of the fcc structure of Pd (JCPDS NO: 87-0641). The reflection at 40.02° (111) is the most intense compared with the other reflections, which indicates the preferred direction for the growth of nanocrystals [37]. A reflection at a 2θ of 33.59° was also observed in addition to the reflections belonging to PdNCs, which may be a result of residual moieties of the leaf extract [38]. The Debye–Scherrer formula was used to estimate the crystalline size of PdNCs:

D = Kλ/β cos θ(1)

where β is the full-width at half maximum of the diffraction peaks, θ is Bragg’s diffraction angle, K = 0.9 is the Scherrer constant or the shape factor, and λ is the wavelength of Cu-Kα. The crystallite size of PdNCs was estimated to be 12.66 nm using the diffraction peak associated with crystal plane (111).

### 2.5. Energy-Dispersive X-Ray Spectroscopy (EDX) and X-Ray Photoelectron Spectroscopy (XPS) Analysis

The purity of the biosynthesized nanoclusters was determined by evaluating their elemental composition via TEM with EDX. The imaging ability of the microscope allowed the selection of the specimen of interest. The EDX spectrum was obtained in terms of the X-ray counts (cps/eV) versus energy (keV). A strong signal for elemental palladium is present in the EDX spectrum, showing the purity of the biosynthesized nanoclusters (Figure 3c). The spectrum also shows a signal for elemental copper because a copper grid was used for sample preparation. The spectrum includes only elemental palladium and copper, with no other elemental signals observed, indicating the purity as well as the synthesis of the palladium nanoclusters. Table S1 lists the detailed EDX analysis results in terms of the elemental compositions, elemental percentages, series, and k factors.

**Figure 4:**
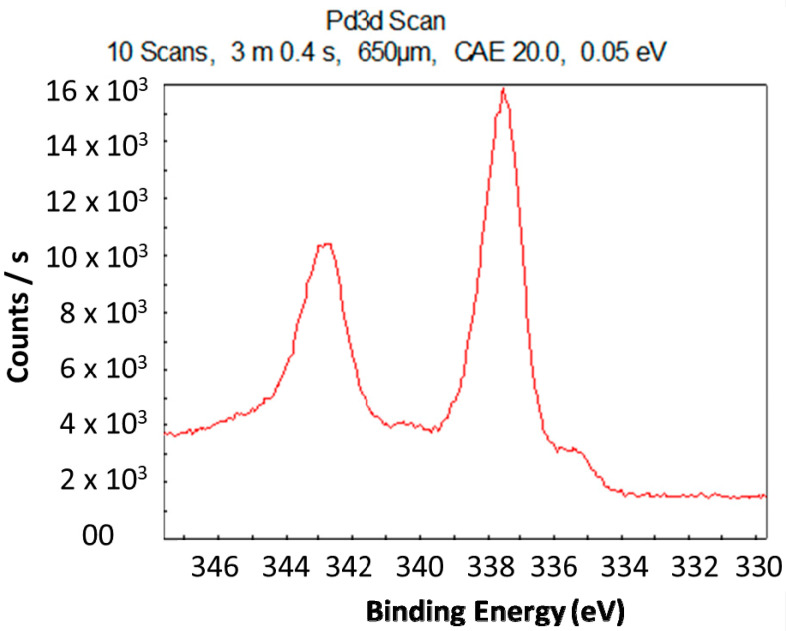
X-ray photoelectron spectroscopy (XPS) spectrum indicating the binding energy of Pd (0).

The oxidation state of the biosynthesized nanoclusters was determined using XPS. The sample for the XPS analysis was prepared by drying the colloidal solution of nanoclusters on a glass plate. The XPS spectrum shows a peak at a binding energy of 335.22 eV, which is the characteristic binding energy of Pd (0) (Figure 4). This clearly indicates that the biosynthesized nanoclusters were zero-valent.

### 2.6. Fourier Transform Infrared (FTIR) Analysis

**Figure 5:**
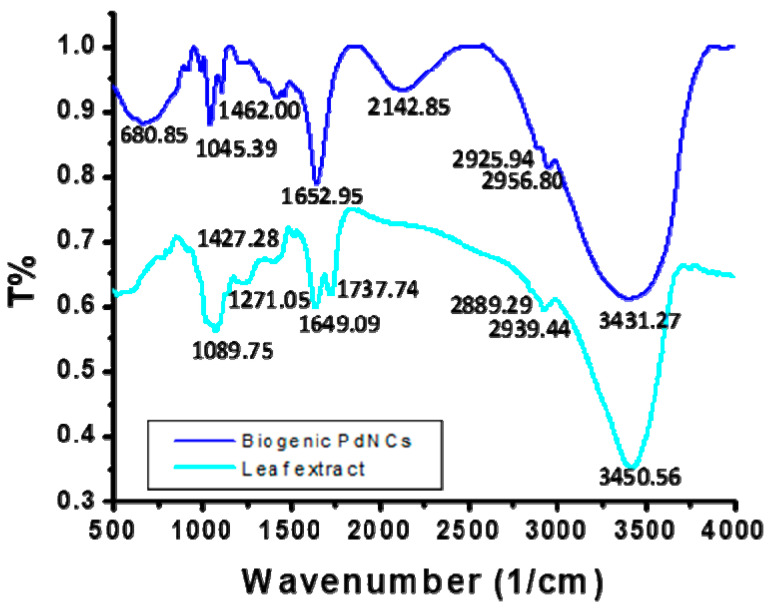
Fourier transform infrared (FTIR) spectra of the *Erigeron Canadensis* L. leaf extract and as-synthesized PdNCs.

The nanoclusters were scanned in the range of 500–4000 cm^−1^ to elucidate the participation of biological molecules originating from the leaf extract (Figure 5). The FTIR spectrum of the leaf extract includes a peak at 3450.56 cm^−1^. This particular peak has broadened and shifted to 3431.27 cm^−1^ in the spectrum of PdNCs, indicating –OH stretching. The leaf extract spectrum shows a peak at 2939.44 cm^−1^, which corresponds to C-H stretching of CH_2_ and CH_3_. This peak has shifted to 2956.80 cm^−1^ in the spectrum of the PdNCs, suggesting the participation of C-H stretching vibration in the biosynthesis of PdNCs. The peak shift between particular functional groups of the leaf extract and nanoclusters indicates a reduction and stabilization of nanoclusters [39].

The peak at 1649.09 cm^−1^ in the leaf extract spectrum shifted to 1652.02 cm^−1^ in the spectrum of the PdNCs, which corresponds to the stretching vibration of COO^−^. The leaf extract spectrum also exhibits a peak at 1450 cm^−1^, which shifted to 1462.0 cm^−1^ in the PdNCs’ spectrum, corresponding to the N-H stretching vibration in the amide linkages of the protein. The peak at 1271.05 cm^−1^ present in the leaf extract spectrum, corresponding to the C-N stretching of amines, is not observed in the PdNCs’ spectrum [40]. The leaf extract and PdNCs’ spectra contain peaks at 1089.75 and 1045.39 cm^−1^, respectively, representing a slight shift. These peaks are analogous to that at 1074 cm^−1^, corresponding to the presence of flavanones adsorbed on the surface of the nanoclusters. This indicated that the structure of flavanones is affected as a result of binding with nanoclusters [39].

### 2.7. Biosynthesis Mechanism

*Erigeron* species has been used to cure indigestion, enteritis, hematuria, and epidemic hepatitis [41]. Erigeron species is a rich source of γ-pyranone derivatives, flavonoids, and phenolic acids [42,43], which play a significant role in the synthesis of nanomaterials. The aqueous solution of *E. Canadensis* L. leaf extract was used as a reducing and capping agent for the synthesis of PdNCs.

The possible biosynthesis mechanism of PdNCs is developed on the basis of UV/Vis spectroscopy, FTIR, XPS, TEM, and EDX. All parts of the plant are rich in polyphenolic compounds, which actively participate in neutralizing the impact of ROS. High antioxidative properties were shown by flavonoids and tannins present in the aqueous extract of the *E. Canadensis* L. leaf. Reduction of Pd^2+^ to Pd^0^ by polyphenolic compounds occurred when the leaf extract was added to the palladium chloride solution. The color of the reaction solution changed as a function of time, which was monitored by UV/Vis spectroscopy (Figure 1). The complete reduction of Pd^2+^ into Pd^0^ took 150 min to incubate. Subsequently, the neutralized Pd^0^ experienced nucleation, which produced significantly small-sized nanoparticles. Finally, these particles took the shape of clusters via non-covalent bonding to stabilize (Figure 2). Furthermore, the biomolecules present in the leaf extract covered the surface of the nanocluster and provided high stability. The FTIR illustrated that a peak at 1649.09 cm^−1^ in the spectrum of the leaf extract shifted to 1652.02 cm^−1^ in PdNCs, which corresponds to the stretching vibration of COO^−^ (Figure 5). Another peak at 1450 cm^−1^ in the leaf extract shifted to 1462.0 cm^−1^ in PdNCs, corresponding to the N-H stretching vibration in the amide linkages of the protein. The peaks at 1089.75 cm^−1^ (leaf extract) and 1045.39 cm^−1^ (PdNCs) are analogous to that at 1074 cm^−1^, corresponding to the presence of flavanones adsorbed on the surface of the nanoclusters. These peaks evidently demonstrate the role of biomolecules in the synthesis of nanoclusters. However, more experimental analyses would be required to understand the comprehensive biosynthesis mechanism.

### 2.8. Effect of PdNCs’ Concentrations

**Figure 6:**
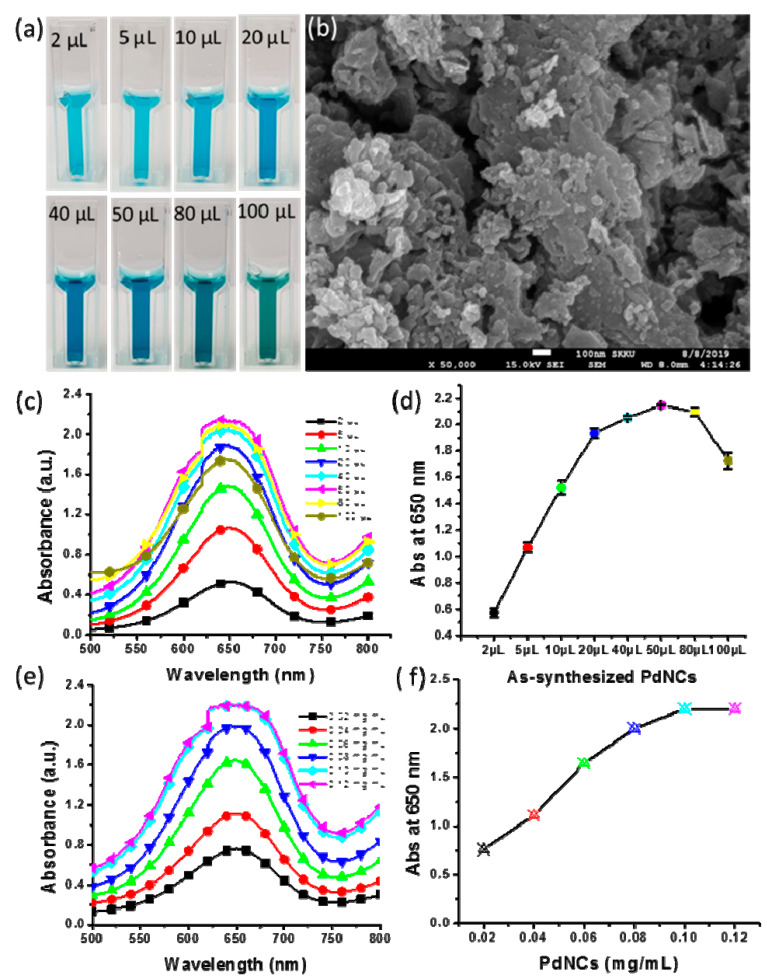
Effect of the nanocluster concentration: (**a**) Cuvettes showing the effect of the nanocluster concentration on the color intensity; (**b**) Scanning electron microscopy (SEM) micrograph of freeze-dried nanoclusters; (**c**) Absorbance spectra for various concentrations of as-synthesized PdNCs; (**d**) Triplicate experiments performed on the effect of varying the concentration of as-synthesized nanoclusters to produce error bars representing the standard deviations; (**e**) Absorbance spectra at various concentrations of freeze-dried nanoclusters; (**f**) Absorbance at 650 nm for assays having various concentrations of freeze-dried nanoclusters.

Additions of as-synthesized nanoclusters of 2–100 µL were considered to analyze the impact of the concentration of PdNCs on the oxidation of TMB. Figure 6a shows that the color intensity of the assay solutions increased with an increase in the concentration of nanoclusters; however, above 50 µL (0.0125 mg/mL) of as-synthesized PdNCs, the color of the assay solutions changed to light green with a blue color. All the samples were scanned with a UV/Vis spectrophotometer to obtain the absorbance spectra for determination of the optimum concentration. Figure 6c shows the spectra for the assays with different concentrations of nanoclusters. The absorbance at 650 nm increased rapidly with increasing concentration of PdNCs; however, with the addition of greater than 20 µL of as-synthesized nanoclusters, the absorbance value increased little up to the addition of 50 µL (Figure 6d). Thereafter, the absorbance decreased with an increase in concentration resulting from the addition of more than 50 µL of PdNCs. We also analyzed the impact of the synthesized nanoclusters using the freeze-dried powder of PdNCs in the concentration range from 0.02 to 12 mg/mL (Figure 6e,f). High concentrations of nanoclusters were applied, but the absorbance was much higher for the case of the as-synthesized nanoclusters with ultralow concentrations. The reason behind the low oxidation of TMB was determined from the SEM micrograph. The micrograph shows that a certain amount of nanoclusters became agglomerated and lost their morphology; however, some amount still retained their small size, as observed in the SEM micrographs (Figure 6b). We thus conclude that a smaller size of nanoclusters with ultralow concentration was effective to achieve high oxidation of TMB. Furthermore, we ran a control assay without H_2_O_2_ and observed that, as the concentration of nanocluster increased, the control assay color intensity also increased because the nanoclusters themselves have a color (Figure S4). Hence, the experimental results indicate that 10 µL (0.0025 mg/mL) of as-synthesized nanoclusters is effective for the oxidation of 0.525 mM TMB.

### 2.9. Selectivity of Assay

**Figure 7:**
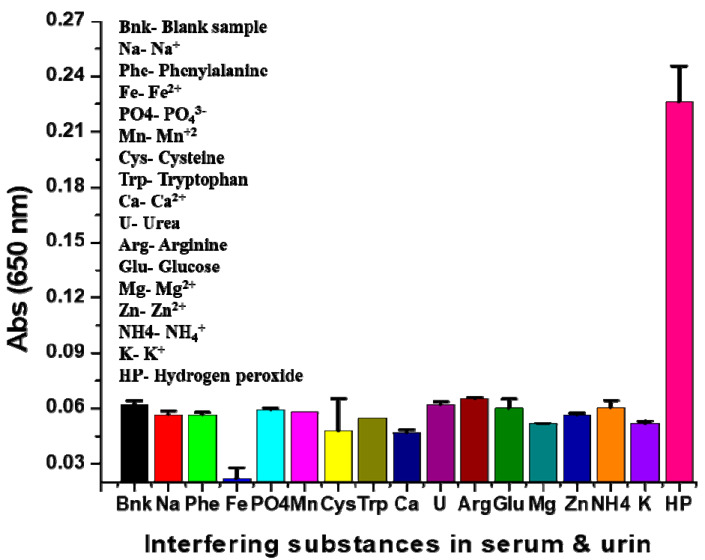
Selectivity of the assay for H_2_O_2_ detection: UV/Vis absorbance at 650 nm of various serum-interfering substances and performed triplicate experiments of the selectivity to produce error bars representing the standard deviations.

The selectivity of the assay was determined by exposing it to other relevant and interfering substances present in serum. The peroxidase mimetic activity of the nanoclusters possesses the specific characteristic of oxidizing TMB (colorless) into oxTMB (blue color) in the presence of H_2_O_2_. This feature of the assay can help easily distinguish H_2_O_2_ from various interfering substances in serum. Various interfering substances (100 μM phenylalanine, cysteine, tryptophan, arginine, glucose, urea, Na^+^, Fe^2+^, PO_4_^3-^, Mn^+2^, Ca^2+^, Mg^2+^, Zn^2+^, NH_4_^+^, and K^+^) were included to evaluate the selectivity of the assay. Each interfering substance was assayed independently; when H_2_O_2_ was present in the sample, the nanoclusters would oxidize the TMB, the color of the assay changed rapidly from colorless to blue, and the UV/Vis spectra exhibited a strong peak at 650 nm. Figure S5 shows that no peak was observed at 650 nm in the case of PdNCs, TMB, and TMB + PdNCs. We also performed the comparative analysis for TMB oxidation between biosynthesized PdNCs and leaf extract. Figure S6 shows that the strong absorbance was found at 650 nm, only with nanocluster. Therefore, the leaf extract did not participate in the oxidation of TMB. The color change was only observed in the assays containing H_2_O_2_; the other interfering substances remained the same color as the blank assay. Figure 7 shows that high absorbance intensity was found only in the assays containing H_2_O_2_ as a strong color developed, whereas other interfering substances present in serum did not develop a blue color. This reveals that the assay is highly selective for H_2_O_2_. A quantitative analysis of the colorimetric assay was performed by comparing the absorbance of the sample and blank (Figure 7). This is significant for the determination of the intensity of the assay color, which represents the quantity of oxidized TMB.

### 2.10. Sensitivity of Assay

**Figure 8:**
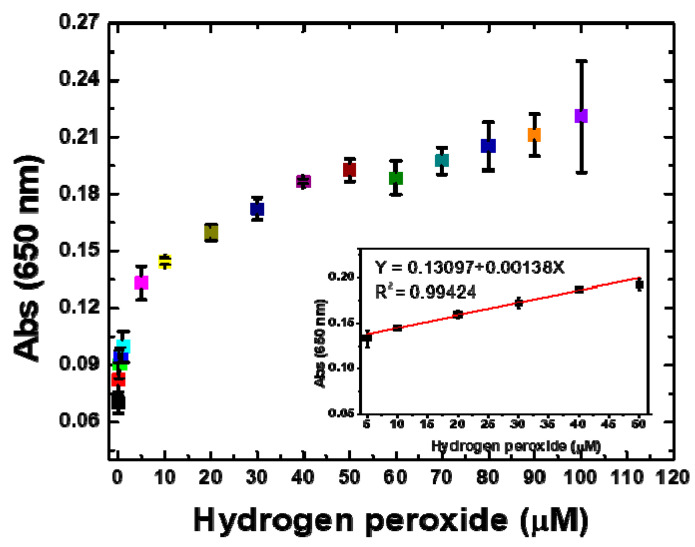
Sensitivity of the assay for H_2_O_2_ detection: UV/Vis absorbance values at 650 nm vs. H_2_O_2_ concentration (0.0625–100 μM); the assay exhibited a linear response at low concentrations.

The significance of the assay depends on its detection sensitivity at a lower concentration of target molecules. The assay sensitivity was evaluated through the detection of ultralow concentrations of H_2_O_2_ in a sample. Various H_2_O_2_ concentrations were used (0.0625–100 μM) to determine the sensitivity by measuring the absorbance of the assay. The existence of H_2_O_2_ in the sample facilitated the oxidation of the chromogenic peroxidase substrate TMB and provided a blue color to the assay owing to the formation of oxTMB. It was observed that the absorbance at 650 nm increased with increasing H_2_O_2_ concentration. All the assays were incubated at room temperature (22 °C) for 20 min, which was determined to be the optimum incubation time. Figure 8 shows that the lowest detected concentration was 0.0625 μM, as the absorbance is higher than that of the blank sample at 650 nm. The color intensity of the assay increased with the increasing concentration of H_2_O_2_, and the absorbance at 650 nm increased with the increasing intensity of the assay color. The oxidation of TMB was quantitatively determined by considering the absorbance; the absorbance at 650 nm versus the H_2_O_2_ concentrations was plotted to quantify the oxidation of TMB (Figure 8). The assay exhibited a linear response at low H_2_O_2_ concentrations of 5 to 50 μM, and a linear regression correlation coefficient of 0.99424 was obtained (Figure 8 inset). The sensitivity response of assay was evaluated up to 100 μM H_2_O_2_. The lowest H_2_O_2_ concentration that could be detected by the naked eye through the assay color was found to be 40 μM. The results show that the proposed method has a good response at lower concentrations and a lower detection limit compared with those of previously reported methods (Table 1). Therefore, the proposed colorimetric assay is an ultrasensitive and selective method.

## 3. Materials and Methods

### 3.1. Materials

Palladium(II) chloride was purchased from Sigma–Aldrich (St. Louis, MO, USA) and used as a precursor for the synthesis of PdNCs. The chromogenic substrate 3,3′,5,5′-tetramethylbenzidine (TMB) was also obtained from Sigma–Aldrich (St. Louis, MO, USA) for evaluation of the peroxidase mimetic activity of PdNCs. Hydrogen peroxide (H_2_O_2_) was purchased from Samchun Chemical Co. Ltd. (Seoul, South Korea). L- arginine, cysteine, L- tryptophan, and L- phenylalanine were purchased from Sigma–Aldrich (St. Louis, MO, USA). All other chemicals, including sodium chloride, iron chloride tetrahydrate, potassium phosphate dibasic, magnesium chloride, calcium chloride, magnesium nitrate hexahydrate, zinc acetate dehydrate, ammonium sulfate, potassium chloride, urea, and glucose, were of analytical-grade and were used as received without further purification. Deionized water was used in all the experiments related to the biosynthesis of nanoparticles and catalytic oxidation of TMB.

### 3.2. Preparation of Leaf Extract

Fresh leaves of *Erigeron Canadensis* L. were collected from the campus of Sungkyunkwan University (Suwon, Gyeonggido, Republic of Korea) (Figure 1a). The leaves were washed several times with distilled water and dried at ambient temperature to remove the water from the surface of the leaves. Then, 8.5 g of chopped leaves was dispersed into 100 mL of deionized water in a 200 mL Erlenmeyer flask and placed on a magnetic stirrer at 500 revolutions per min (rpm) while the dispersion was boiled. Subsequently, the aqueous solution was allowed to cool at room temperature and was filtered through Whatman filter paper, yielding the required leaf extract. The extract was stored at 4 °C for use in the biosynthesis of PdNCs.

### 3.3. Biosynthesis of Nanoclusters

Palladium chloride (2.5 mM) was dissolved in 20 mL of deionized water and placed on a magnetic stirrer for 1 h at ambient temperature and 600 rpm; the magnetic stirrer temperature was maintained at 85 °C. Then, 1 mL of the as-prepared aqueous solution of the leaf extract was added drop by drop, followed by stirring under the same conditions for 180 min. The bio-reduction of Pd(II) to Pd(0) was clearly indicated by a color change from light yellow-brown to a dark brownish color. The biosynthesized nanoclusters were analyzed by measuring the absorbance of the colloidal solution as a function of time.

### 3.4. Characterization of Nanoclusters

The biosynthesized PdNCs were characterized by ultraviolet-visible (UV/Vis) spectroscopy (UH-5300, Hitachi, Japan) in the scanning range of 300–800 nm. The size distribution profile of the PdNCs was analyzed via dynamic light scattering (DLS; Zetasizer Nano S90, Malvern). Fourier transform infrared (FTIR) spectroscopy (FTS 7000, Varian, Australia) was performed to elucidate the role of biological molecules in the synthesis of nanoclusters. The morphology, size, d-spacing (inter-atomic spacing), and crystalline nature of the PdNCs were characterized via transmission electron microscopy (TEM; JEM-3010, JEOL, Japan). Meanwhile, the elemental composition of the nanoclusters was identified via energy-dispersive X-ray spectroscopy (EDX). The X-ray diffraction patterns were determined with an X-ray Diffractometer (X’Pert PRO, PANanalytical, Netherland) with CuKα radiation (λ = 1.5417 Å). The freeze-dried nanoclusters were further analyzed using scanning electron microscopy (SEM; Zeiss, EVO 18, Germany). The oxidation state of the PdNCs was evaluated using X-ray photoelectron spectroscopy (XPS; Thermo Scientific, UK).

### 3.5. Colorimetric Detection

The peroxidase mimetic activity of as-synthesized PdNCs was investigated through the catalytic oxidation of the peroxidase substrate TMB. The working solution of H_2_O_2_ was freshly prepared by diluting H_2_O_2_ (34.5%/d- 1.135) with deionized water. In a typical reaction (total volume: 1 mL), 0.525 mM (5 μL) of TMB (stock solution prepared in DMSO), 20 mM (up to 50 μL from stock) of H_2_O_2_, and 10 μL of as-synthesized PdNCs were combined; the final volume was reached by adding 0.1 M acetate buffer. The assay solution was incubated for 20 min at room temperature (22 °C). The TMB-H_2_O_2_ chromogenic reaction was evaluated by measuring the absorption peak at 650 nm.

### 3.6. Effect of PdNCs’ Concentration

In these experiments, the concentration of a colloidal solution of as-synthesized PdNCs varied in the range of 2 to 100 μL (0.0005–0.025 mg/mL). The reaction was performed in a total volume of 1 mL composed of 0.525 mM (5 μL) TMB (stock solution prepared in DMSO), 20 mM (50 μL from stock) H_2_O_2_, and varying concentration of as-synthesized nanoclusters. The freeze-dried powder of the nanoclusters was also used to analyze the impact of concentration. A range of powder samples (0.02–12 mg/mL) was added to the reaction. The obtained results were compared to determine the conditions for the nanoclusters in terms of their stability and concentration impact.

### 3.7. Selectivity of Assay

The selectivity of the assay against various serum-interfering substances was investigated using 100 μM of phenylalanine, cysteine, tryptophan, arginine, glucose, urea, Na^+^, Fe^2+^, PO_4_^3−^, Mn^+2^, Ca^2+^, Mg^2+^, Zn^2+^, NH_4_^+^, and K^+^. The color change of the assay was examined with respect to H_2_O_2_ visually. The assay was also analyzed by measuring its absorption from 500 to 800 nm by UV/Vis spectrophotometry. A quantitative analysis of the oxidation of the chromogenic substance TMB was performed by comparing the absorbance values at 650 nm.

### 3.8. Sensitivity of Assay

The sensitivity of the assay was determined by determining the lowest detectable concentration of H_2_O_2_. The concentration of nanoclusters in the colloidal solution was estimated by freeze drying the colloidal solution; however, as-synthesized nanoclusters were applied in the assay. The working solution of H_2_O_2_ was freshly prepared by diluting H_2_O_2_ (34.5%/d- 1.135) in deionized water. In a typical reaction (total volume 1 mL), 0.525 mM TMB (5 μL from a stock solution prepared in DMSO), varying concentrations of H_2_O_2_ (not more than 100 μL from stock), and 10 μL (0.0025 mg/mL) of as-synthesized PdNCs were combined; the remaining volume was filled by adding 0.1 M acetate buffer (pH 5). The concentrations of H_2_O_2_ for the determination of the sensitivity of the colorimetric assay ranged from 0.0625 to 100 μM. A quantitative analysis of the H_2_O_2_ was performed by measuring the absorbance using a UV/Vis spectrophotometer. All the assays were incubated for 20 min at room temperature (22 °C).

## 4. Conclusions

In this study, we developed an unprecedented biological method for the synthesis of PdNCs using *E. Canadensis* L. leaf extract. This is the first report of the biological synthesis of nanoclusters. The Z-average diameter of the synthesized nanoclusters was found to be 64.47 nm by DLS, whereas a diameter of 55 nm was obtained by TEM because the DLS analysis showed the hydrodynamic size of the nanostructures. The synthesized nanoclusters were found to be significantly stable with a narrow size distribution. Nanoclusters were demonstrated to possess peroxidase mimetic activity that could oxidize a 3,3′,5,5′-tetramethylbenzidine (TMB) peroxidase substrate. The assay was shown to exhibit a sufficient signal at an ultralow concentration of 0.0625 µM H_2_O_2_. Therefore, the developed assay provides an easy-to-use platform for the detection of H_2_O_2_ for clinical purposes.

## Figures and Tables

**Table 1:** Comparison of various reported methods for H_2_O_2_ detection.

Detection Mode	Detection Limit	Operating Time	Ref.
Gold nanoparticle-based colorimetric biosensor assay	1 µM	15 min	[20]
Fe_2_(MoO_4_)_3_ micromaterial-MMT-based colorimetric assay	0.7 µM	400 s	[21]
Au nanocluster-based fluorescence assay	0.2 µM	8 min	[22]
Magnetic mesoporous silica nanoparticle-based colorimetric assay	4.0 µM	20 min	[23]
MnO_2_ nanosheet-modified UCNP-based fluoroimmunoassay	0.9 µM	40 min	[24]
Ag_2_S–MMT-based colorimetric assay	19.16 µM	21 min	[25]
Fluorescent NS–carbon quantum dots	4.0 µM	10 min	[26]
TPE-BO-based fluoroimmunoassay	0.52 µM	15 min	[27]
CD-NP-BE-based fluoroimmunoassay	0.5 µM	60 min	[28]
Silver nanoparticles for colorimetric detection	0.09 µM	60 min	[29]
Ag-coated TFBG-SPR assay	0.2 µM	20 min	[30]
Silver nanoparticle-based colorimetric assay	5.0 µM	40 min	[31]
Bimetallic metal-organic framework of the type MOF (Co/2Fe)	5.0 µM		[32]
ZnO nanoparticles	50 µM		[33]
Biosynthesized palladium nanocluster-based colorimetric assay	0.0625 µM	20 min	

## Supplementary Materials

The following are available online at https://www.mdpi.com/1420-3049/25/15/3349/s1, Figure S1: Size distribution profile of biosynthesized nanoclusters, Figure S2. Various magnifications of TEM micrographs of PdNCs obtained after sonication of colloidal solution of nanoclusters for assessing their stability, Figure S3. DLS obtained after sonication of colloidal solution of nanoclusters for assessing their stability, Figure S4. Results for the control assay (without H_2_O_2_) showing absorbance at 650 nm in response to the addition of varying amounts of as-synthesized nanoclusters. Inset cuvettes show impact of nanocluster concentration on intensity of color without H_2_O_2_, Figure S5. Absorbance spectra of 0.525 mM TMB, 10 µL nanoclusters, and the combination of both; the inset cuvettes show the color, Figure S6. Comparative analysis for TMB oxidation between biosynthesized PdNCs and leaf extract; Table S1: Elemental compositions of PdNCs obtained with EDX analysis.

## References

[1] Genet J.-P. (2003). Asymmetric catalytic hydrogenation. Design of new ru catalysts and chiral ligands: From laboratory to industrial applications. Acc. Chem. Res..

[2] Behrens M., Studt F., Kasatkin I., Kühl S., Hävecker M., Abild-Pedersen F., Zander S., Girgsdies F., Kurr P., Kniep B.-L. (2012). The active site of methanol synthesis over Cu/ZnO/Al_2_O_3_ industrial catalysts. Science.

[3] Huang Y., Ren J., Qu X. (2019). Nanozymes: Classification, catalytic mechanisms, activity regulation, and applications. Chem. Rev..

[4] Tripathi R.M., Ahn D., Kim Y.M., Chung S.J. (2020). Enzyme mimetic activity of zno-pd nanosheets synthesized via a green route. Molecules.

[5] Liang M., Fan K., Pan Y., Jiang H., Wang F., Yang D., Lu D., Feng J., Zhao J., Yang L. (2013). Fe_3_O_4_ magnetic nanoparticle peroxidase mimetic-based colorimetric assay for the rapid detection of organophosphorus pesticide and nerve agent. Anal. Chem..

[6] Darabdhara G., Sharma B., Das M.R., Boukherroub R., Szunerits S. (2017). Cu-ag bimetallic nanoparticles on reduced graphene oxide nanosheets as peroxidase mimic for glucose and ascorbic acid detection. Sens. Actuators B Chem..

[7] Chen W.H., Vázquez-González M., Kozell A., Cecconello A., Willner I. (2018). Cu^2+^-modified metal–organic framework nanoparticles: A peroxidase-mimicking nanoenzyme. Small.

[8] Wang X., Zhao X. (2009). Contribution of oxidative damage to antimicrobial lethality. Antimicrob. Agents Chemother..

[9] Nita M., Grzybowski A. (2016). The role of the reactive oxygen species and oxidative stress in the pathomechanism of the age-related ocular diseases and other pathologies of the anterior and posterior eye segments in adults. Oxidative Med. Cell. Longev..

[10] Finkel T., Serrano M., Blasco M.A. (2007). The common biology of cancer and ageing. Nature.

[11] Inoguchi T., Li P., Umeda F., Yu H.Y., Kakimoto M., Imamura M., Aoki T., Etoh T., Hashimoto T., Naruse M. (2000). High glucose level and free fatty acid stimulate reactive oxygen species production through protein kinase C--dependent activation of NAD(P)H oxidase in cultured vascular cells. Diabetes.

[12] Andersen J.K. (2004). Oxidative stress in neurodegeneration: Cause or consequence?. Nat. Med..

[13] Park L., Zhou P., Pitstick R., Capone C., Anrather J., Norris E.H., Younkin L., Younkin S., Carlson G., McEwen B.S. (2008). Nox2-derived radicals contribute to neurovascular and behavioral dysfunction in mice overexpressing the amyloid precursor protein. Proc. Natl. Acad. Sci. USA.

[14] Tripathi R.M., Park S.H., Kim G., Kim D.H., Ahn D., Kim Y.M., Kwon S.J., Yoon S.Y., Kang H.J., Chung S.J. (2019). Metal-induced redshift of optical spectra of gold nanoparticles: An instant, sensitive, and selective visual detection of lead ions. Int. Biodeterior. Biodegrad..

[15] Tripathi R., Yoon S.-Y., Ahn D., Chung S.J. (2019). Facile synthesis of triangular and hexagonal anionic gold nanoparticles and evaluation of their cytotoxicity. Nanomaterials.

[16] Sriramulu M., Sumathi S. (2018). Biosynthesis of palladium nanoparticles using saccharomyces cerevisiae extract and its photocatalytic degradation behaviour. Adv. Nat. Sci. Nanosci. Nanotechnol..

[17] Wang W., Zhang B., Liu Q., Du P., Liu W., He Z. (2018). Biosynthesis of palladium nanoparticles using shewanella loihica PV-4 for excellent catalytic reduction of chromium (VI). Environ. Sci. Nano.

[18] Tripathi R., Chung S.J. (2020). Reclamation of hexavalent chromium using catalytic activity of highly recyclable biogenic Pd (0) nanoparticles. Sci. Rep..

[19] Tripathi R.M., Kumar N., Bhadwal A.S., Gupta R.K., Shrivastav B.R., Shrivastav A. (2015). Facile and rapid biomimetic approach for synthesis of hap nanofibers and evaluation of their photocatalytic activity. Mater. Lett..

[20] Lin W.Z., Yeung C.Y., Liang C.K., Huang Y.H., Liu C.C., Hou S.Y. (2018). A colorimetric sensor for the detection of hydrogen peroxide using DNA-modified gold nanoparticles. J. Taiwan Inst. Chem. Eng..

[21] Wang B., Ju P., Zhang D., Han X., Zheng L., Yin X., Sun C. (2016). Colorimetric detection of H_2_O_2_ using flower-like Fe_2_(MoO_4_)_3_ microparticles as a peroxidase mimic. Microchim. Acta.

[22] Zong C., Wang M., Li B., Liu X., Zhao W., Zhang Q., Liang A., Yu Y. (2017). Sensing of hydrogen peroxide and glucose in human serum via quenching fluorescence of biomolecule-stabilized au nanoclusters assisted by the fenton reaction. RSC Adv..

[23] Wang Y., Zhou B., Wu S., Wang K., He X. (2015). Colorimetric detection of hydrogen peroxide and glucose using the magnetic mesoporous silica nanoparticles. Talanta.

[24] Yuan J., Cen Y., Kong X.J., Wu S., Liu C.L., Yu R.Q., Chu X. (2015). MnO_2_-nanosheet-modified upconversion nanosystem for sensitive turn-on fluorescence detection of H_2_O_2_ and glucose in blood. ACS Appl. Mater. Interfaces.

[25] Liu Q., Jiang Y., Zhang L., Zhou X., Lv X., Ding Y., Sun L., Chen P., Yin H. (2016). The catalytic activity of ag2s-montmorillonites as peroxidase mimetic toward colorimetric detection of H_2_O_2_. Mater. Sci. Eng. C.

[26] Singh V.K., Yadav P.K., Chandra S., Bano D., Talat M., Hasan S.H. (2018). Peroxidase mimetic activity of fluorescent ns-carbon quantum dots and their application in colorimetric detection of H_2_O_2_ and glutathione in human blood serum. J. Mater. Chem. B.

[27] Zhang W., Liu W., Li P., Huang F., Wang H., Tang B. (2015). Rapid-response fluorescent probe for hydrogen peroxide in living cells based on increased polarity of C–B bonds. Anal. Chem..

[28] Wu G., Zeng F., Yu C., Wu S., Li W. (2014). A ratiometric fluorescent nanoprobe for H_2_O_2_ sensing and in vivo detection of drug-induced oxidative damage to the digestive system. J. Mater. Chem. B.

[29] Zong C., Li B., Wang J., Liu X., Zhao W., Zhang Q., Nie X., Yu Y. (2018). Visual and colorimetric determination of H_2_O_2_ and glucose based on citrate-promoted H_2_O_2_ sculpturing of silver nanoparticles. Microchim. Acta.

[30] Zhang X., Wu Z., Liu F., Fu Q., Chen X., Xu J., Zhang Z., Huang Y., Tang Y., Guo T. (2018). Hydrogen peroxide and glucose concentration measurement using optical fiber grating sensors with corrodible plasmonic nanocoatings. Biomed. Opt. Express.

[31] Zhang L., Li L. (2016). Colorimetric detection of hydrogen peroxide using silver nanoparticles with three different morphologies. Anal. Methods.

[32] Yang H., Yang R., Zhang P., Qin Y., Chen T., Ye F. (2017). A bimetallic (Co/2Fe) metal-organic framework with oxidase and peroxidase mimicking activity for colorimetric detection of hydrogen peroxide. Microchim. Acta.

[33] Sodzel D., Khranovskyy V., Beni V., Turner A.P., Viter R., Eriksson M.O., Holtz P.O., Janot J.M., Bechelany M., Balme S. (2015). Continuous sensing of hydrogen peroxide and glucose via quenching of the UV and visible luminescence of ZnO nanoparticles. Microchim. Acta.

[34] Danaei M., Dehghankhold M., Ataei S., Hasanzadeh Davarani F., Javanmard R., Dokhani A., Khorasani S., Mozafari M.R. (2018). Impact of particle size and polydispersity index on the clinical applications of lipidic nanocarrier systems. Pharmaceutics.

[35] Malvern Instruments Ltd. (2011). Inform White Paper Dynamic Light Scattering.

[36] Stolyarov I.P., Gaugash Y.V., Kryukova G.N., Kochubei D.I., Vargaftik M.N., Moiseev I.I. (2004). New palladium nanoclusters. Synthesis, structure, and catalytic properties. Russ. Chem. Bull..

[37] Li X., Goh T.W., Xiao C., Stanton A.L., Pei Y., Jain P.K., Huang W. (2016). Synthesis of monodisperse palladium nanoclusters using metal–organic frameworks as sacrificial templates. ChemNanoMat.

[38] Tripathi R.M., Gupta R.K., Bhadwal A.S., Singh P., Shrivastav A., Shrivastav B.R. (2015). Shrivastav. Fungal biomolecules assisted biosynthesis of Au–Ag alloy nanoparticles and evaluation of their catalytic property. IET Nanobiotechnol..

[39] Abboud Y., Eddahbi A., El Bouari A., Aitenneite H., Brouzi K., Mouslim J. (2013). Microwave-assisted approach for rapid and green phytosynthesis of silver nanoparticles using aqueous onion (*Allium cepa*) extract and their antibacterial activity. J. Nanostruct. Chem..

[40] Jyoti K., Baunthiyal M., Singh A. (2016). Characterization of silver nanoparticles synthesized using urtica dioica linn. Leaves and their synergistic effects with antibiotics. J. Radiat. Res. Appl. Sci..

[41] Li X., Yang M., Han Y.-F., Gao K. (2005). New sesquiterpenes from erigeron annus. Planta Med..

[42] Nazaruk J., Kalemba D. (2009). Chemical composition of the essential oils from the roots of *Erigeron acris* L. and *Erigeron annuus* (L.) pers. Molecules.

[43] Tripathi R., Chung S.J. (2019). Biogenic nanomaterials: Synthesis, characterization, growth mechanism, and biomedical applications. J. Microbiol. Methods.

