# Supplementary material for: Phytosynthesis of Palladium Nanoclusters: An Efficient Nanozyme for Ultrasensitive and Selective Detection of Reactive Oxygen Species
Source: Molecules. 2020 Jul 23;25(15):3349. doi: 10.3390/molecules25153349 (PMC7436022; doi:10.3390/molecules25153349)
Supplement: Supplementary file 1 [file molecules-25-03349-s001.pdf]

molecules

MDPI

Article

Phytosynthesis of palladium nanoclusters: An efficient nanozyme for ultrasensitive and selective detection of reactive oxygen species

Ravi Mani Tripathi [1,2], and Sang J. Chung [1,*]

[1] School of Pharmacy, Sungkyunkwan University, 2066 Seoburo, Jangan-gu, Suwon, Gyeonggido 16419, Korea.

[2] Amity Institute of Nanotechnology, Amity University Uttar Pradesh, Sector 125, Noida 201303, India

* Correspondence: sjchung@skku.edu; Tel.: +82-31-290-7703; Fax +82-31-292-8800

Received: date; Accepted: date; Published: date

Table S1. Elemental compositions of PdNCs obtained with EDX analysis.

Element	Line Type	k Factor	k Factor type	Absorption Correction	Wt%	Wt% Sigma	Atomic %
Cu	K series	1.233		0.50	28.39	0.33	39.90
Pd	L series	1.809		0.60	71.61	0.33	60.10
Total:					100.00		100.00

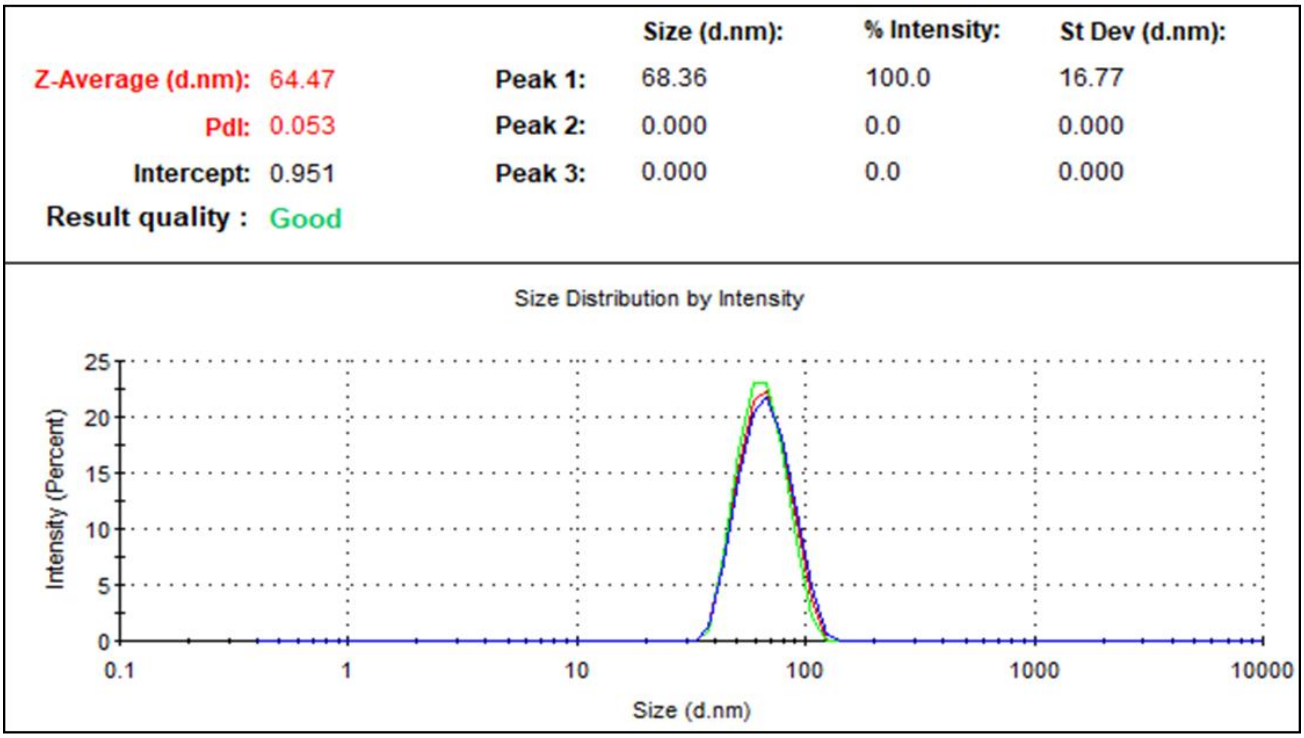

Figure S1. Size distribution profile of biosynthesized nanoclusters.

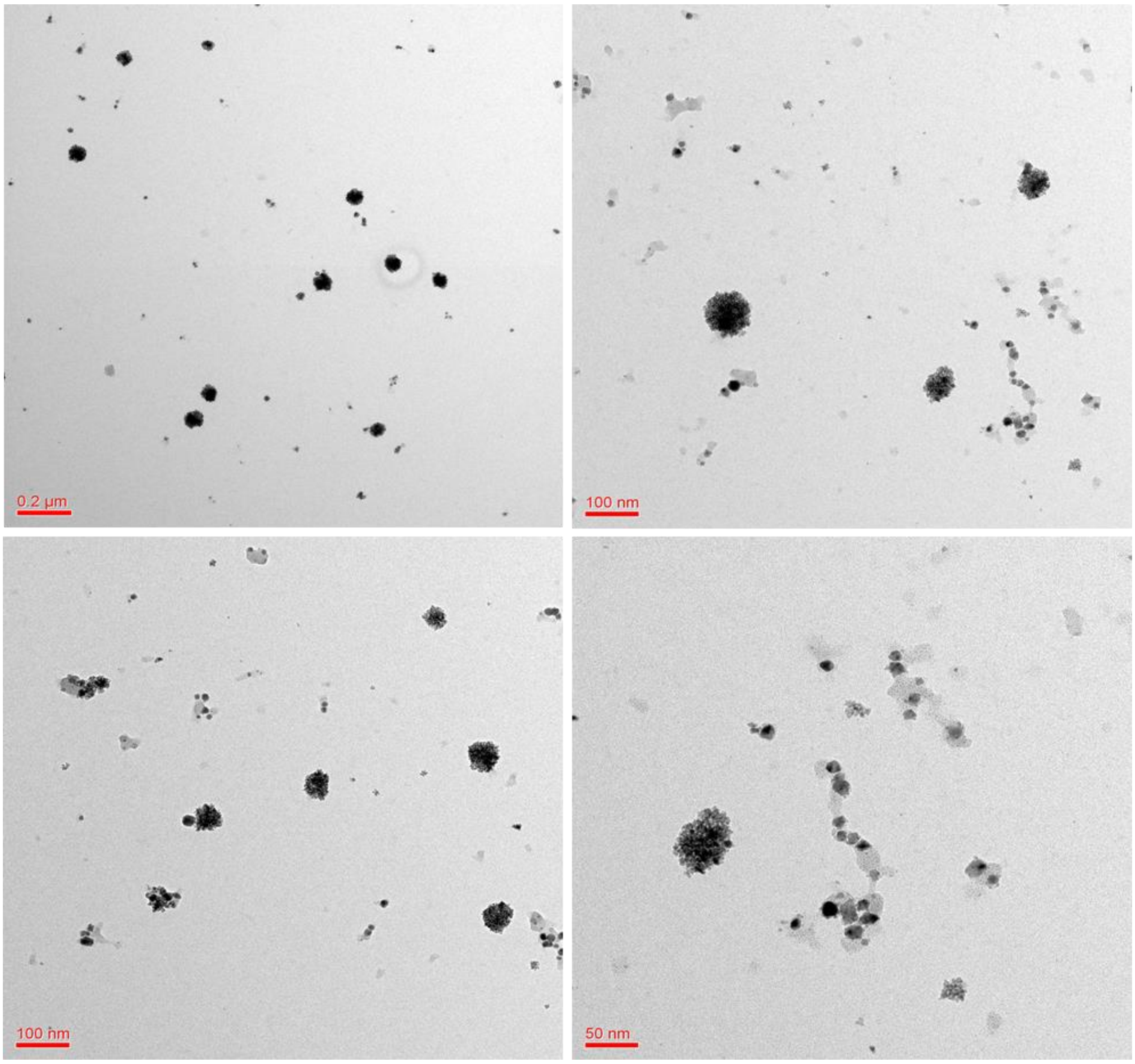

Figure S2. Various magnifications of TEM micrographs of PdNCs obtained after sonication of colloidal solution of nanoclusters for assessing their stability.

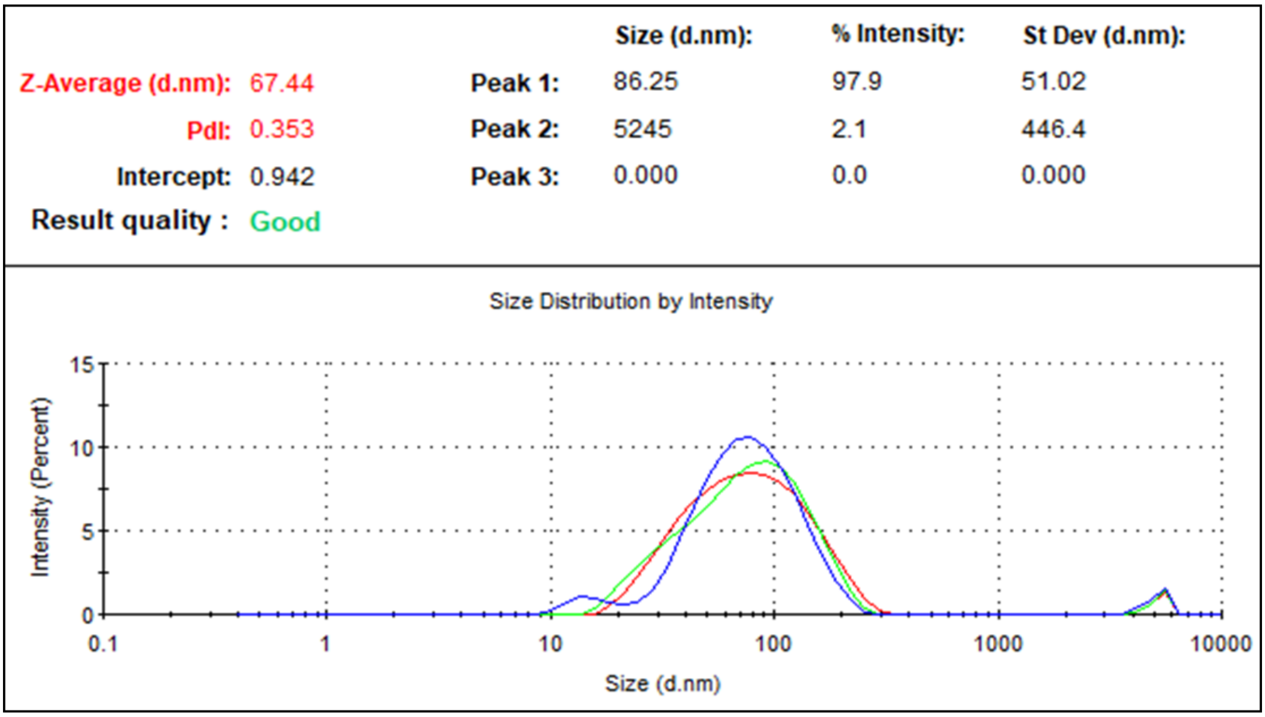

Figure S3. DLS obtained after sonication of colloidal solution of nanoclusters for assessing their stability.

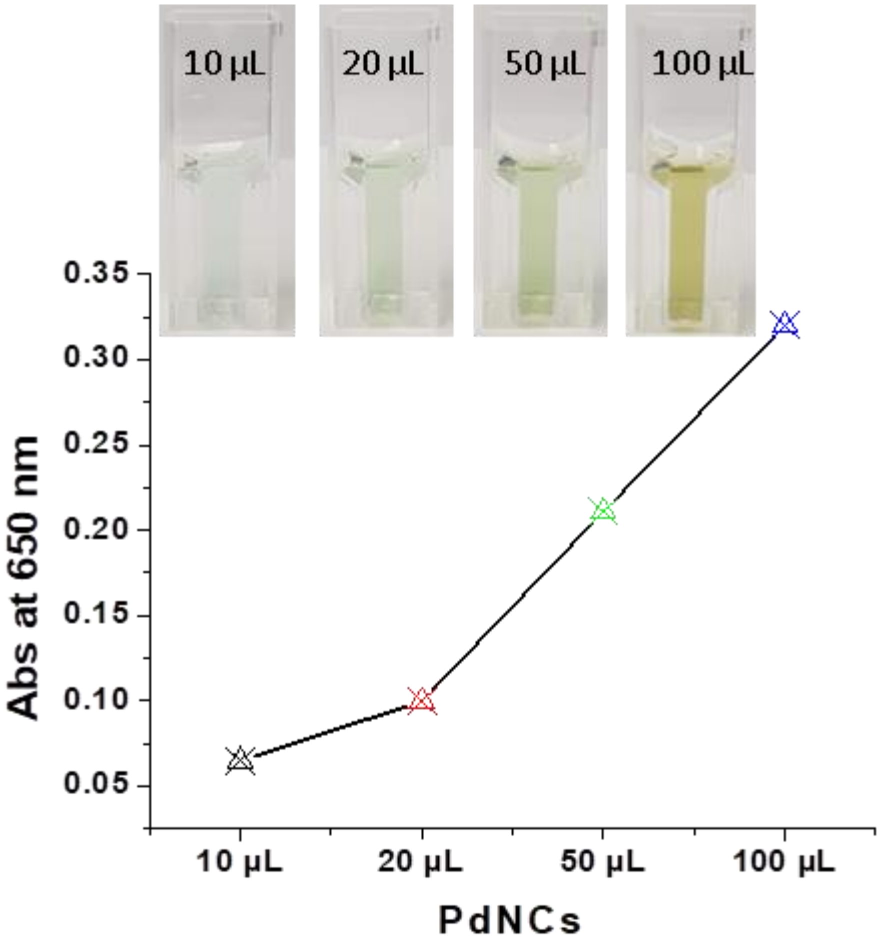

Figure S4. Results for the control assay (without H_2O_2) showing absorbance at 650 nm in response to the addition of varying amounts of as-synthesized nanoclusters. Inset cuvettes show impact of nanocluster concentration on intensity of color without H_2O_2.

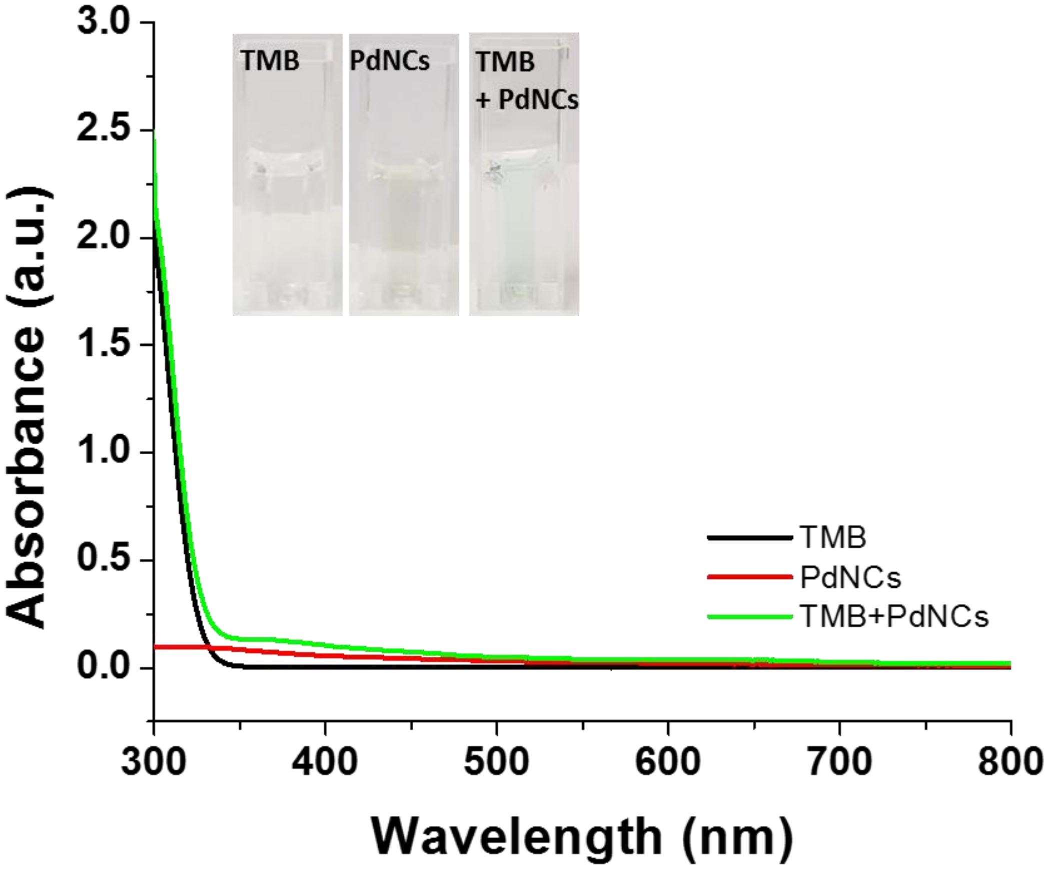

Figure S5. Absorbance spectra of 0.525 mM TMB, 10 μL nanoclusters, and the combination of both; the inset cuvettes show the color.

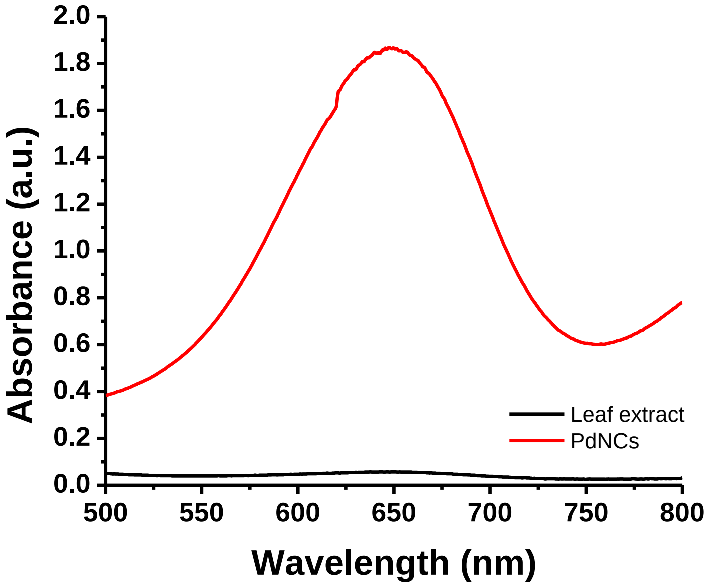

Figure S6. Comparative analysis for TMB oxidation between biosynthesized PdNCs and leaf extract.
